# Percutaneous Double‐Loaded All‐Suture Knotless Anchor at the 6‐O'Clock Position for Shoulder Instability

**DOI:** 10.1002/atn2.70018

**Published:** 2026-04-29

**Authors:** Benjamin Sanderford, Kelly Wolfe, Brandon Pham, Bennett Janeski, Guy Ball

**Affiliations:** ^1^ McLaren Oakland Pontiac Michigan U.S.A.; ^2^ Lake Erie College of Osteopathic Medicine Erie Pennsylvania U.S.A.

## Abstract

Glenohumeral instability is a common condition encountered by orthopaedic sports surgeons, particularly in young, active athletes. Initial management is nonoperative, consisting of physical therapy, but some patients may still require surgical intervention. Arthroscopic stabilization, via capsulorrhaphy or plication, remains the primary operative approach. Here, we describe our technique, which utilizes an Arthrex double‐loaded, knotless 2.6 mm FiberTak soft anchor placed at the 6‐o'clock position for labral and capsular repair.

## Graphical abstract

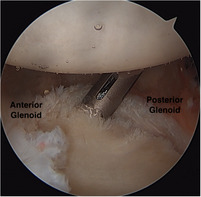

**VIDEO 1:** Video with narration describing the placement of the Arthrex 2.6 FiberTak double‐loaded soft anchor at the 6‐o’clock position and the repair of the inferior labrum and two bands of the inferior glenohumeral ligament. Viewing the patient's right side, in the lateral decubitus position throughout the entirety of the video. Anterior Portal: 0:04‐1:57; 3:01‐End of video Posteior Portal: 1:57‐3:00. Video content can be viewed at https://doi.org/10.1002/atn2.70018.

Given the inherently concave and shallow morphology of the glenoid fossa, the glenohumeral joint confers the greatest range of motion of any articulation in the human body; however, this increased mobility concomitantly predisposes the shoulder to biomechanical instability.^1^ Although traumatic dislocations frequently result in acute labral detachments, other etiologies—such as pan‐labral tears, superior labrum anterior and posterior tears, and multidirectional instability—also compromise joint integrity. Initial management consists of physical therapy with dynamic stabilization. However, in refractory cases, surgical intervention is required to restore functional stability.^1^, ^2^

Arthroscopic capsulolabral stabilization remains the mainstay of operative techniques aiming to tighten the surrounding soft tissue and recreate stability. Recurrent dislocations, however, remain a significant concern.^1^, ^3^ Shibata et al. identified several patient factors that increase the risk of redislocations, including Hill‐Sachs defects and critical glenoid bone loss.^3^ In addition, surgical technique, such as the use of fewer than 4 anchors, may also contribute to redislocation. However, anchor‐related complications, such as knot stacking and ensuing frictional arthritis, also exist.^4^, ^5^, ^6^

Although the literature on optimal anchor placement remains limited, Kim et al. showed improved labral height and Rowe scores in patients whose anchors were placed on the glenoid face rather than at the edge.^7^ Owens et al. have shown that the addition of a 6‐o'clock anchor to the conventional 3‐, 4‐, and 5‐o'clock positions biomechanically enhances peak resistance to anterior translation.^8^ Furthermore, advancements in current technology, such as a double‐loaded suture anchor, can assist with issues such as glenoid bone loss.^9^ Recurrent dislocations, knot stacking leading to frictional arthritis, and lack of ideal literature on anchor placement underscore the need for optimized fixation strategies. We describe a technique for placing a double‐loaded 2.6 mm all‐suture anchor at the 6‐o'clock position through a percutaneously placed 7‐o'clock portal.

## SURGICAL TECHNIQUE

### Patient Evaluation

Evaluating a patient for shoulder instability begins with a thorough history and full shoulder examination. It is essential to characterize the details of a single traumatic instability event or atraumatic multidirectional instability. Shoulder examination should include apprehension testing, load and shift, and sulcus sign, as well as assessing Beighton's criteria.

Standard radiographs are obtained, which include anteroposterior, Grashey, scapula Y, and axillary views. Additional views include West Point view for possible glenoid bone loss and Stryker view for evaluation of a Hill‐Sachs lesion. A computed tomography scan can address the amount and displacement of glenoid bone loss if present. Magnetic resonance imaging, with or without arthrogram, is beneficial for evaluating labral tears and other associated injuries, including but not limited to humeral avulsion of the glenohumeral ligament, a glenoid articular defect, and anterior labral periosteal sleeve avulsions.

Selection of surgical versus nonsurgical management requires a thorough discussion of the risks and benefits of all options. Ultimately, the final treatment plan should align with the patient's individual goals.

### Procedure

This procedure is performed arthroscopically using a standard posterior viewing portal, anteroinferior and anterosuperior working portals, and a percutaneous 7‐o'clock portal.

### Positioning and Equipment

The patient is placed in the lateral decubitus position using a bean bag with all bony prominences appropriately padded The operative arm is draped and placed into a STaR sleeve (Arthrex, Naples, FL) and hooked up to the Shoulder Traction Tower (Arthrex, Naples, FL) attached to the Clark Rail of a traditional slide top table. This distracts the humeral head away from the glenoid to allow for working space around the labrum and capsule. A 30‐degree arthroscope was utilized throughout the procedure. Standard sterile technique was maintained.

### Arthroscopic Technique

Landmarks are identified and marked. A standard posterior viewing portal is established first. An initial diagnostic arthroscopy is performed. Under direct visualization, an anteroinferior portal is created with a spinal needle just over the superior edge of the subscapularis tendon through the rotator interval. An 8.25 mm twist‐in cannula is placed. A spinal needle is once again used to create a second portal high in the rotator interval just below the biceps tendon. A 7.0 mm twist‐in cannula is placed. At this time, the camera is removed, and through the scope sheath, a switching stick is placed. The scope sheath is removed and the camera is moved to the high anterior portal. A 7.0 twist‐in cannula is placed overtop of the switching stick. The camera is moved back to the posterior portal.

The arthroscopic shaver is introduced through the high anterior cannula and loose debris is removed from the shoulder.

A labral elevator is used to float the labrum from the 2‐o'clock position to the 7‐o'clock position. The labrum is elevated until the subscapularis muscle fibers are visualized under the floated labral tissue.

With the use of the arthroscopic burr or shaver, the anterior glenoid is prepared for the labral repair.

**FIGURE 1:**
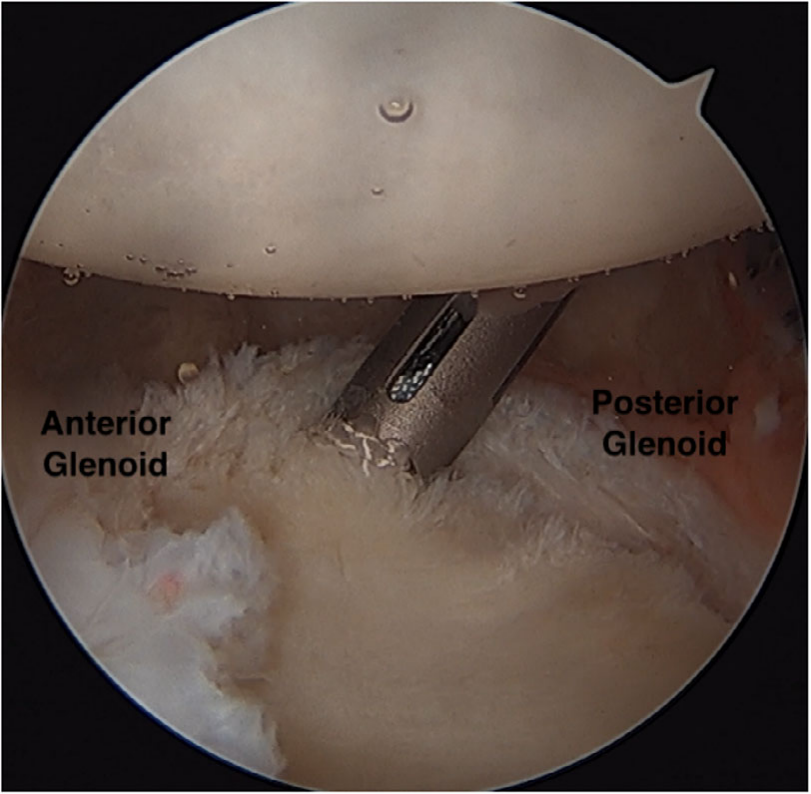
Right side, lateral decubitus position. The arthroscope is placed at the high anterior viewing portal. The straight cannula is placed through the 7‐o'clock percutaneous portal and placed at the 6‐o'clock position of the glenoid in order to place the anchor.

At this time, a percutaneous 7‐o'clock portal is created using a 14‐gauge spinal needle followed by the nitinol wire through the spinal needle. Over the top of the nitinol wire, a dilator is able to be passed, and finally, the percutaneous straight cannula for the double‐loaded 2.6 FiberTak soft anchor (Arthrex) can be placed at the 6‐o'clock position roughly 4 to 5 mm onto the glenoid face (Figure 1). The drill is passed through the cannula and drilled to a positive stop. Through the cannula, the anchor is placed and malleted into place. The anchor is set by pulling all the suture limbs simultaneously.

**FIGURE 2:**
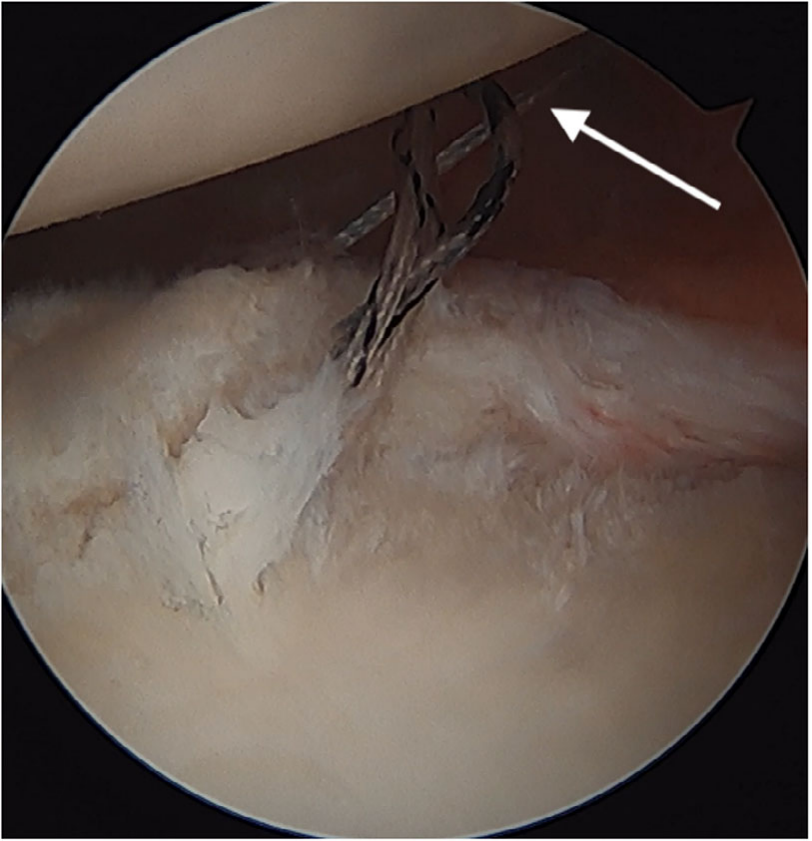
Right side, lateral decubitus position. Viewing from the high anterior portal. The first repair stitch (white arrow) pierces through the posterior labrum and posterior band of the inferior glenohumeral ligament as it courses back through the posterior portal. The remaining sutures are parked at the 7‐o'clock percutaneous portal.

**FIGURE 3:**
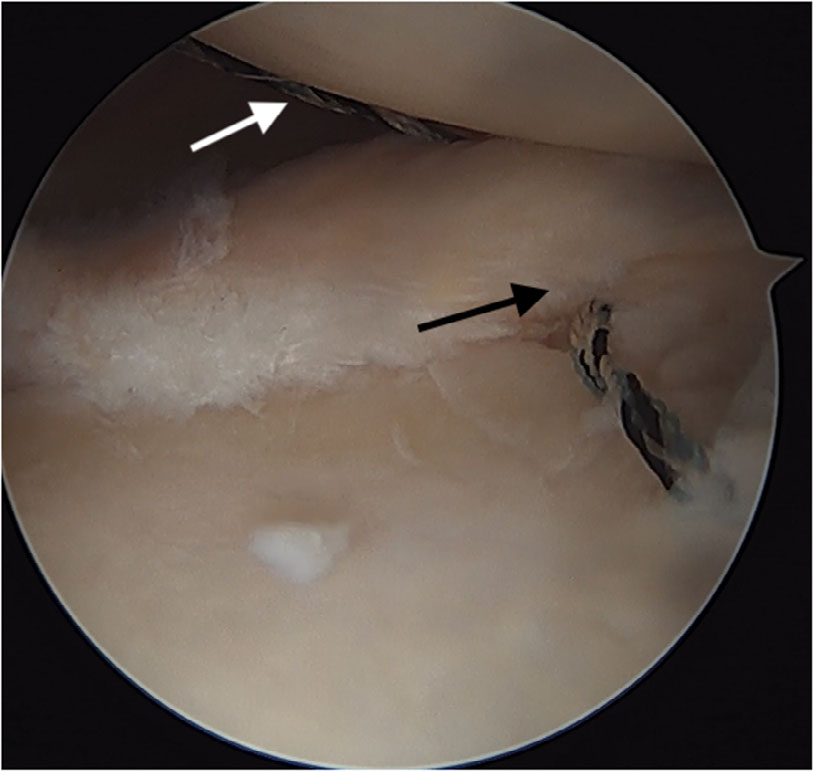
Right side, lateral decubitus position. Viewing from the posterior portal. The second repair suture (white arrow) is seen piercing through the anterior labrum (black arrow) and the anterior band of the inferior glenohumeral ligament.

**FIGURE 4:**
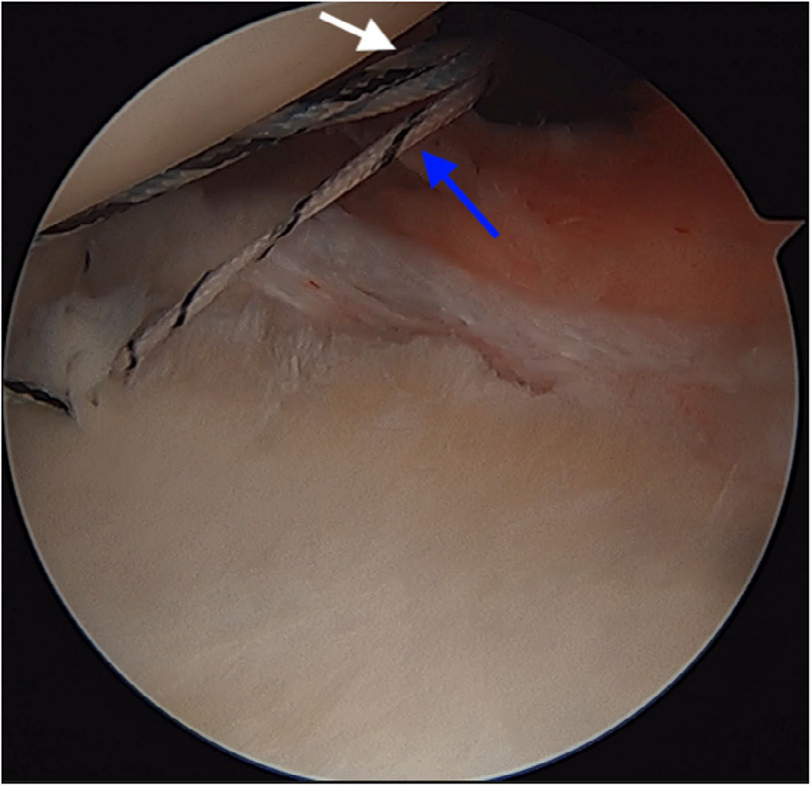
Right side, lateral decubitus position. Viewing from the high anterior portal. The two repair sutures (white arrow) and the shuttling stitch (blue arrow) are being parked out of the posterior portal to prep are the repair sutures for conversion.

**FIGURE 5:**
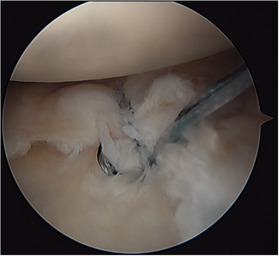
Right side, lateral decubitus position. The final repair is seen with the anchor at the 6‐o'clock position and the 2 repair sutures grabbing the inferior labrum both anteriorly and posteriorly, as well as the anterior and posterior bands of the inferior glenohumeral ligament.

A curved suture passer is placed through the posterior portal. The suture passer is used to pierce the posterior band of the inferior glenohumeral ligament (pIGHL) and labral tissue. The nitinol wire is pushed into the joint and pulled out the posterior portal. A ring grasper is passed through the posterior portal, and the most posterior of the 2 repair stitches is grasped and pulled out the portal. The nitinol suture passing wire can now be used to pull the repair stitch through the pIGHL and labral tissue (Figure 2). The camera is now moved to the posterior portal. The opposite direction curved suture passer is now passed through the low anterior portal and the anterior band of the inferior glenohumeral ligament (aIGHL) and labral tissue. The nitinol wire is grasped with an arthroscopic grasper and parked out the high anterior portal. With the use of a ringed grasper through the high anterior portal, the anterior repair stitch is grasped and pulled out of the cannula. The nitinol wire is now able to be used to pass the repair stitch through the aIGHL and labral tissue (Figure 3). Both repair stitches and the looped limb of the passing stitch through the anchor are grabbed with a ring grasper and pulled out the posterior portal (Figure 4). The 2 repair stitches are passed through the shuttling stitch and converted by pulling on the limb of the shuttling stitch that remains out the percutaneous portal. The anterior and posterior limbs can now be sequentially tightened, reducing the aIGHL and pIGHL and creating a true inferior capsular shift. The sutures can be left out the percutaneous portal and retensioned at the end of the case. The final repair can be seen in Figure 5 with the anchor at the 6‐o'clock position and the 2 repair sutures grabbing the inferior labrum both anteriorly and posteriorly, as well as the anterior and posterior bands of the inferior glenohumeral ligament (IGHL).

The camera is moved back to the posterior portal and the anterior labral repair can now be completed by any technique desired. Our preference is with 3 to 4 Arthrex knotless 1.8 mm FiberTak Soft Anchors placed along the anterior glenoid rim. The described technique can be seen in Video 1.

### Postoperative Care

The patient is placed into an abduction arm sling for 6 weeks. Patients may begin passive range of motion with physical therapy starting at 6 weeks following surgery. Active‐assisted and active range of motion, along with limited isometric strengthening, is initiated at 8 weeks. Full strengthening occurs at 12 weeks. Patients typically return to normal daily activities by approximately 5 months postoperatively. Return to sport is individualized based on the specific demands of the sport and results of provocative testing. The typical return to sport testing begins after 4 months once full active range of motion has returned and once operative upper‐extremity strength is symmetrical.

## DISCUSSION

It has been well established that arthroscopic Bankart repair is a safe and effective method for treating anterior shoulder instability.^1^, ^2^ New surgical techniques and devices are constantly being developed to improve functional outcome scores, diminish recurrence, and promote a safe return to sport for athletes.^6^, ^7^, ^9^ It has also been well established that anchor placement at the 6‐o'clock position improves the stability of Bankart repair.^3^, ^7^, ^8^ This article outlines a technique utilizing a 2.6 mm knotless, double‐loaded anchor placed at the 6‐o'clock position. We believe this technique provides numerous benefits. First, knotless anchors allow for less interdependent variability among surgeons. Burkhart et al. performed a study showing the vast variability of arthroscopic knots in terms of consistency and strength among expert arthroscopists.^10^ Multiple other studies have also shown the consistency, potential biomechanical improvements, and reduced surgical times provided by knotless versus knotted anchors.^11^, ^12^, ^13^ Second, this double‐loaded anchor allows for repair of both aIGHL and pIGHL, providing an effective capsular shift with controlled tension. Third, the knotless mechanism prevents knot stacking at the inferior glenoid, reducing the risk of irritation, and theoretical frictional arthritis.^4^, ^14^

Anterior shoulder stabilization procedures have been postulated to fail for several reasons. However, it is often believed that suboptimal inferior capsular shift is the culprit. The IGHL is the primary restraint in the position of vulnerability with the arm in maximal abduction and external rotation. Therefore, failure to adequately shift the inferior capsule and IGHL does not effectively address the primary stabilizer of the shoulder in its most vulnerable position. One of the main reasons for an inadequate shift is due to difficulty accessing the 6‐o'clock position. The use of a percutaneous portal for this technique is a distinct advantage in accessing the inferior glenoid face. It further minimizes the need to lever on the humeral head to access the inferior glenoid through a standard high or low anterior portal. Another advantage of this technique is the incorporation of a knotless fixation system. Knot tying at the 6‐o'clock position is technically demanding and can result in the formation of 1 or more knot stacks, which in turn may increase the risk of frictional changes on the humeral head. Although the clinical significance of this risk has been debated, eliminating knot stacks altogether represents a theoretical but meaningful advantage. The specific Arthrex 2.6 mm FiberTak anchor we use in this technique has the advantage of being double‐loaded. At the time the lead surgeon implemented this technique, no other company offered a similar product. The distinct advantage of this anchor is a low‐profile all‐suture anchor with the ability to shift both the anterior and posterior bands through a single anchor. See Table 1 for advantages and disadvantages.

**TABLE 1:** List of Advantages and Disadvantages of the Described Technique

Advantages	Disadvantages
**Direct Access to the Inferior Glenoid Face** • The percutaneous portal allows improved access to the 6‐o'clock position. • Minimizes the need to lever on the humeral head when compared with standard anterior portals.	**Portal Access Challenges** • Establishing a 7‐o'clock percutaneous portal can be difficult, especially in patients with larger arms.
**Knotless Fixation System** • Avoids the technical difficulty and variability of knot tying. • Prevents knot stack formation, which may cause frictional changes on the humeral head. • Eliminates even the theoretical risk of knot‐induced articular damage.	**Suture Management Issues** • Limited portals and cannulas increase the risk of sutures tangling or looping on themselves. • Requires careful management with a ring grasper before deploying the knotless mechanism.
**Double‐Loaded Implant design** • Low‐profile, all‐suture design. • Double‐loaded, allowing stabilization of both anterior and posterior bands through a single anchor. • Unique offering at the time of adoption (no comparable alternative available).	
**Ease of Adoption** • Considered relatively straightforward to learn and implement.	

Despite these advantages, the technique is not without limitations. Achieving a percutaneous portal at the 7‐o'clock position can be difficult, particularly in patients with larger arms. As with any surgical method, there is an inherent learning curve; however, we believe this approach is straightforward to adopt. Suture management represents another challenge, as limited portals and cannulas make it difficult to manage the repair stitch and shuttling stitch. The primary pitfall arises when sutures become entangled and knot, although this can be mitigated by running each suture with a ring grasper prior to deploying the knotless mechanism. An unavoidable limitation to the technique is the combined tensioning mechanism of the anchor. Although it would be ideal if each limb could be passed separately and tensioned individually, the suture limbs can be sequentially tightened despite being passed simultaneously through a single anchor body. All pitfalls and pearls can be seen in Table 2.

**TABLE 2:** List of All Pitfalls and Pearls of the Described Technique

Pitfalls	Pearls
Larger arms may create difficulty with portals	Longer spinal needles in the perc kit are helpful with larger arms
Sutures can become knotted and lead to deployment issues	Use a ring grasper to run the sutures to mitigate entanglement
Failure to fully elevate the inferior labrum can limit the effectiveness of the inferior shift	

## DISCLOSURES

The authors (B.S., K.W., B.P., B.J., G.B.) declare that they have no known competing financial interests or personal relationships that could have appeared to influence the work reported in this article.
